# Exploring Sustainable Agriculture with Nitrogen-Fixing Cyanobacteria and Nanotechnology

**DOI:** 10.3390/molecules29112534

**Published:** 2024-05-28

**Authors:** Taufiq Nawaz, Liping Gu, Shah Fahad, Shah Saud, Bruce Bleakley, Ruanbao Zhou

**Affiliations:** 1 Department of Biology/Microbiology, South Dakota State University, Brookings, SD 57007, USA; 2 Department of Agronomy, Abdul Wali Khan University Mardan, Mardan 23200, KP, Pakistan; 3 College of Life Science, Linyi University, Linyi 276000, China

**Keywords:** plant-nanoparticle interaction, nitrogen-fixing cyanobacteria, stress tolerance, plants, agricultural nanotechnology

## Abstract

The symbiotic relationship between nitrogen-fixing cyanobacteria and plants offers a promising avenue for sustainable agricultural practices and environmental remediation. This review paper explores the molecular interactions between nitrogen-fixing cyanobacteria and nanoparticles, shedding light on their potential synergies in agricultural nanotechnology. Delving into the evolutionary history and specialized adaptations of cyanobacteria, this paper highlights their pivotal role in fixing atmospheric nitrogen, which is crucial for ecosystem productivity. The review discusses the unique characteristics of metal nanoparticles and their emerging applications in agriculture, including improved nutrient delivery, stress tolerance, and disease resistance. It delves into the complex mechanisms of nanoparticle entry into plant cells, intracellular transport, and localization, uncovering the impact on root-shoot translocation and systemic distribution. Furthermore, the paper elucidates cellular responses to nanoparticle exposure, emphasizing oxidative stress, signaling pathways, and enhanced nutrient uptake. The potential of metal nanoparticles as carriers of essential nutrients and their implications for nutrient-use efficiency and crop yield are also explored. Insights into the modulation of plant stress responses, disease resistance, and phytoremediation strategies demonstrate the multifaceted benefits of nanoparticles in agriculture. Current trends, prospects, and challenges in agricultural nanotechnology are discussed, underscoring the need for responsible and safe nanoparticle utilization. By harnessing the power of nitrogen-fixing cyanobacteria and leveraging the unique attributes of nanoparticles, this review paves the way for innovative, sustainable, and efficient agricultural practices.

## 1. Introduction

Molecular nitrogen (N_2_) makes up most of the atmosphere on earth and accounts for around 78% of the total gas volume. Despite its abundance, most living things cannot use gaseous nitrogen directly [1]. To overcome this limitation, certain microorganisms have evolved the ability to perform biological nitrogen fixation (BNF), converting atmospheric nitrogen into bioavailable forms such as ammonia and other nitrogen compounds [2]. To maintain the finite supply of biologically accessible nitrogen, BNF is a crucial ecological mechanism [3,4]. Primary productivity in a variety of ecosystems, including terrestrial and aquatic environments, as well as symbiotic relationships with plants, depend on this process [5]. Among the numerous BNF-capable species, nitrogen-fixing cyanobacteria play a crucial role in delivering fixed nitrogen into the biosphere [6].

The phylum of prokaryotic microbes known as cyanobacteria, or blue-green algae, has a spectacular evolutionary history that dates back around 2.7 billion years [7,8,9]. Earth’s atmosphere was shaped by these ancient bacteria because they were among the first to use photosynthesis, in which oxygen is released as a byproduct of metabolic activity [10,11,12]. Cyanobacteria occur in many different habitats and exhibit a wide range of morphologies, including unicellular, filamentous, and even colonial forms [13,14,15]. Nitrogen-fixing cyanobacteria carry out solar-powered BNF independently as free-living organisms, in contrast to higher plants that depend on symbiotic relationships with rhizobia for nitrogen fixation [16]. They have specialized cell structures termed heterocysts, which are oxygen-limited environments where nitrogen fixation occurs [17,18]. The ability of cyanobacteria to sequester oxygen-sensitive nitrogenase enzymes within heterocysts allows for effective nitrogen fixation while guarding against deactivation of the enzymes by oxygen [6,19,20]. Due to their exceptional traits and ecological importance, nitrogen-fixing cyanobacteria have drawn considerable attention for their potential applications in sustainable agriculture and environmental remediation [21]. Harnessing the nitrogen-fixing abilities of cyanobacteria could lead to reduced dependence on synthetic fertilizers, mitigating environmental pollution from excessive fertilizer usage [22]. Additionally, their potential to establish beneficial symbiotic associations with crop plants holds promise for enhancing crop yield and overall agricultural productivity [23]. Chemical fertilizers have traditionally been widely used to supplement nitrogen requirements in agriculture, resulting in increasing agricultural yields and sustaining the expanding human population [24]. Eutrophication, greenhouse gas emissions, and water pollution are some of the environmental issues that have been brought up by the overuse of synthetic fertilizers. In this situation, it is crucial to adopt sustainable methods that preserve agricultural production while reducing dependency on chemical fertilizers [25].

Agriculture is one of the many scientific disciplines particularly interested in the emerging science of nanotechnology. A particle is a tiny entity that functions as a unit in nanoscience or nanotechnology in terms of its properties and transportation. Therefore, one of the factors utilized to categorize such particles is the particle diameter. According to a convention, coarse particles are defined between 10,000 and 2500 nm in size, fine particles as those between 2500 and 100 nm in size, and nanoparticles (NPs) between 1 and 100 nm in size, whether they are dispersed in gaseous, liquid, or solid media [26] differ from their bulk counterparts in terms of specific physicochemical characteristics. These properties have allowed them to be used in a wide variety of farming practices, bringing potential benefits but also difficulties that require careful consideration.

Particularly in recent years, metal nanoparticles have shown promise for modernizing farming methods and advancing sustainable crop production. Silver (Ag), zinc oxide (ZnO), copper oxide (CuO), and iron oxide (Fe_2_O_3_) are metal nanoparticles with exceptional antibacterial, antioxidant, and catalytic characteristics. They can also be synthesized into precise shapes and sizes, which increases their adaptability and potential agricultural uses [27]. *Scaridia galli*, a common intestinal parasite in poultry, could be fought off using secondary metabolites from medicinal plants. The study examined the utilization of several secondary metabolites, such as alkaloids, flavonoids, tannins, saponins, and terpenes, found in diverse medicinal plants, as natural substitutes for synthetic medications for treating parasitic diseases in chickens [28]. To assess the potential of nanoplastics for environmental harm, particularly the toxicity of polystyrene nanoparticles to microalgae. The studies were carried out to determine the impact of various polystyrene nanoparticle concentrations on the development of a specific microalgae species. To analyze the toxicity of the nanoparticles, they evaluated variables like growth rate, biomass, chlorophyll content, and cell viability. The study’s findings showed that the growth and viability of microalgae were negatively impacted by exposure to polystyrene nanoparticles. Higher concentrations of nanoparticles resulted in slower growth rates, lower biomass production, less chlorophyll, and more cell death. The results imply that aquatic ecosystems, particularly microalgae populations, may be threatened by nanoplastics like polystyrene nanoparticles [29,30]. Various types of carbon nanoparticles are discussed, including fullerenes, graphene, carbon nanotubes, carbon nanofibers, and black carbon. It divulges details regarding their compositions, dimensions, and characteristics. The chapter also discusses many approaches, including chemical and physical ones, for synthesizing nanoparticles. Evaporation-condensation, spark discharge, and thermal breakdown are examples of physical processes; metallic precursors, reducing agents, and stabilizing agents are examples of chemical approaches. The section shows the benefits and difficulties of each synthesis technique [31,32,33].

The primary goals of using metal nanoparticles in agriculture are to increase agricultural yield as well as plant growth and nutrient uptake. These nanoparticles can have molecular interactions with plant systems, altering cellular functions and favorably affecting plant metabolism [34,35,36,37,38,39]. The targeted distribution of nutrients, hormones, and agrochemicals to plants is made possible by the role of metal nanoparticles as nanocarriers, which optimizes resource use and minimizes environmental effects [40,41]. Metal nanoparticles have enormous potential for use in agriculture; however, their possible detrimental effects on the environment and human health also raise questions. It is possible that the distinctive physicochemical characteristics of nanoparticles, which make them useful for a variety of applications, may also be to blame for their heightened reactivity and potential toxicity. To ensure the safe and sustainable application of metal nanoparticles, it is crucial to understand their fate and behaviors in agricultural ecosystems [42]. Numerous industries have been transformed by nanotechnology, which has provided ground-breaking solutions in fields like electronics, health, and environmental remediation [43]. Nanoparticles (NPs), one of the most promising developments, have drawn significant interest because of their distinctive physicochemical characteristics and possible uses [44]. These characteristics make NPs exceedingly desirable for a variety of applications, including improved imaging [45], improved catalytic activity, and targeted drug administration [46]. Although there is no denying the potential advantages of NPs, widespread acceptance of them has been significantly hampered by concerns about their safety and environmental impact [47]. Potential dangers may also be exacerbated by the distinct physicochemical characteristics of NPs that make them desirable for applications [48]. For example, due to their small size, NPs can easily overcome biological barriers and pose a risk to human health when they come into contact with living beings [47]. Additionally, the high surface area-to-volume ratio of NPs may increase reactivity and increase the risk of toxicity [49].

A detailed comprehension of NPs’ characteristics and behaviors in various situations is necessary due to the complex interaction between their advantages and hazards [50]. Effective risk assessment requires a thorough analysis of the toxicity profiles, fate, and transit of NPs in multiple environmental compartments [51]. To develop strategies to minimize potential hazards while maximizing the benefits of NPs, a multidisciplinary approach involving regulatory authorities, toxicologists, environmental scientists, and nanotechnology specialists is required [52]. Due to the growing global population and increasing demand for food, there is a need to increase agricultural production while ensuring sustainable agricultural practices [53]. In this context, metal nanoparticles (MNPs), which are at the cutting edge of research in the field of improving plant growth, have proven to be a promising way to solve these problems [54]. Due to their distinct physicochemical characteristics and high surface area-to-volume ratio, metal nanoparticles have a wide range of potential applications in agriculture [55,56]. The size, shape, and surface functionalization of MNPs can be changed through the controlled synthesis process, which affects how they interact with plants and their environment [57]. Additionally, MNPs have natural antibacterial characteristics that can aid in reducing plant diseases caused by various pathogens [58]. MNPs have therefore demonstrated potential as a greener substitute for traditional agrochemicals, reducing environmental impact and encouraging sustainable agriculture [1,59].

MNPs have great potential for use in agriculture, but it is important to assess their possible effects on the environment and their long-term consequences for soil health. To understand the benefits and potential hazards of MNP use in agriculture, a thorough evaluation of the available studies on their effects on plant growth enhancement and sustainability is required [60].

## 2. Nanoparticle Uptake and Distribution in Plants

The use of nanoparticles (NPs) in agricultural practices has opened up new opportunities to improve crop productivity and sustainability. Due to their unique physicochemical characteristics and high surface area-to-volume ratio, NPs are endowed with novel capabilities beyond conventional agricultural methods [61]. For this reason, NPs have been designed and manufactured to tackle certain agricultural issues such as nutrient deficit, pest control, and soil degradation. However, a thorough comprehension of NPs’ interactions in plants at the molecular, cellular, and physiological levels is necessary for their use in agriculture. The study of NP absorption, transport, and distribution within different plant tissues is crucial to this understanding [62].

A variety of factors influence the complicated process by which plants absorb NPs. The size, shape, surface charge, and composition of NPs, as well as how they interact with plant surfaces and cellular membranes, all depend on their physicochemical properties. These factors together affect how much and how quickly NPs are taken up by cells, with smaller NPs and those with positive charges frequently exhibiting enhanced cellular internalization [63].

**Figure 1:**
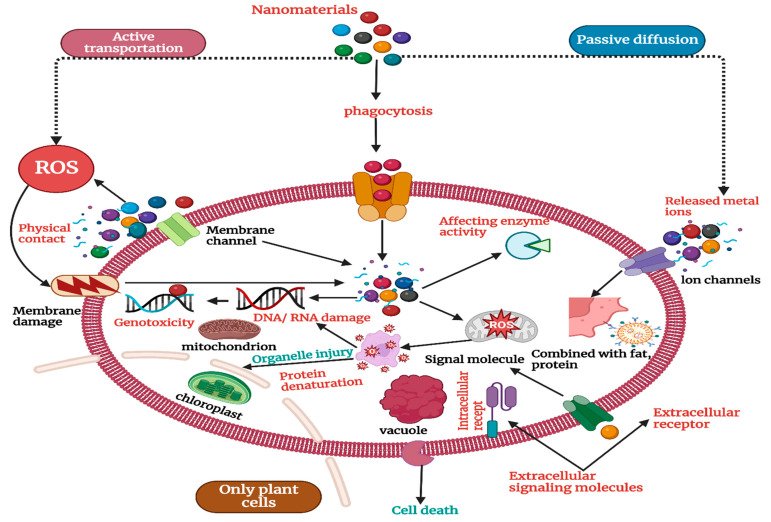
Mechanism of the toxic effects of nanomaterials on soil organisms.

The variance in NP uptake efficiency is further influenced by changes in plant species and genotypes, highlighting the complex interplay between plant-specific features and NP properties [64]. By affecting root architecture and physiology, environmental factors such as soil pH, nutrient availability, and organic matter content can significantly regulate NP uptake. NPs in soil can produce toxic effects at the levels of genes, cells, individual organisms, and communities [65]. As shown in Figure 1, NPs can enter cells through active transport and phagocytosis to induce oxidative stress, affect enzyme activity, damage DNA/RNA, and, in other ways, produce cell toxicity. NPs that do not enter cells can cause membrane damage through physical contact, and metal ions released by metal NPs enter cells mainly through passive diffusion to produce toxic effects [66]. It is believed that ROS production is the main mechanism leading to cell damage. Nanoparticles can stimulate the ground-state oxygen in organisms to produce a large number of ROS, such as hydroxyl radical (·OH), singlet oxygen (^1^O_2_), (O_2_^−^_1_), hydrogen peroxide (H_2_O_2_), etc. [67]. ·OH has a strong oxidation capacity with an oxidation potential of 2.8 V. It can irreversibly destroy many biological macromolecules, including sugars, fats, and proteins [68]. ^1^O_2_ can oxidize and damage a variety of biological components and destroy organisms. O_2_^−^_1_ is unstable and can be converted into •OH and ^1^O_2_ in biological systems [69]. It can cause an imbalance of the REDOX state in the cell, resulting in oxidative damage to the organism, such as cell inflammation and death [70]. A comprehensive understanding of NP absorption mechanisms that goes beyond passive diffusion and includes endocytic pathways is required due to the complicated interactions between these components [71].

After internalization, NPs move through the complex network of plant tissues in simplified and apoplectic pathways. Movement through plasmodesmata, which are nanoscale channels connecting adjacent plant cells, is a component of symplastic transport [72]. As an alternative, NPs can move through the extracellular matrix, which consists of cell walls and intercellular voids, to move via the apoplastic space. The cell wall features, the specific developmental stage of the plant, and the physicochemical properties of NPs all influence the selection of the transport route [73]. Rapid NP movement inside the plant is facilitated by symplastic transport mediated by endocytic vesicles and actin-based movement; however, apoplastic transport may be hampered by physical barriers like the Casparian strip in the root endodermis [74].

The effectiveness of NP uptake is significantly influenced by the root architecture of plants. In particular, root tips, lateral roots, and hairs play a key role in mediating NP interactions with the rhizosphere [75].

The complex variety of organic chemicals found in root exudates affects NP behavior at the soil-root interface and can either increase or decrease NP uptake [76]. By developing symbiotic interactions that improve nutrient and water intake, mycorrhizal linkages further modify NP uptake. According to the available reports, the bryophyte-cyanobacteria symbionts can be divided into three classes according to the bryophyte taxa. Namely, tomentosum-cyanobacteria symbiosis, liverwort-cyanobacteria symbiosis, and bryophyte-cyanobacteria symbiosis. So far, it is known that 41 moss species from 35 genera are capable of symbiotic nitrogen fixation with cyanobacteria (Table 1), which represents only a small part of the moss species known worldwide. Mycorrhizal networks can make it easier for NPs to migrate from hyphal networks to plant roots, potentially affecting how they are later transported through the plant [77].

### 2.1. Mechanisms of Nanoparticle Entry into Plant Cells

In recent years, a lot of research has been done on the relationship between nanoparticles and plant cells [78]. Nanoparticles are attractive candidates for use in agriculture and environmental remediation because they have distinctive physicochemical features that allow them to interact with biological systems in unexpected ways [79]. Their capacity to infiltrate plant cells is one essential factor that controls the effective application of NPs in plant systems. Optimizing the transport and targeting of nanoparticles in plants requires an understanding of the mechanisms underlying their entrance [80].

To assimilate nanoparticles, plant cells have developed different pathways, each involving unique molecular and cellular processes. One of the possible ways in which plant cells can internalize NPs is through endocytosis, a well-studied process in mammalian cells [81]. One or more endocytic mechanisms, including clathrin-mediated endocytosis, caveolin-dependent endocytosis, and clathrin-independent endocytosis, have been proposed to be involved in nanoparticle uptake [82]. Other routes, such as direct passage of nanoparticles through the plasma membrane, have also been proposed [83]. The physical and chemical characteristics of nanoparticles are crucial in defining how they interact with plant cells [84]. The methods of nanoparticle entrance are greatly influenced by surface charge, size, shape, and surface functionalization [85]. For instance, it has been demonstrated that positively charged nanoparticles exhibit greater cellular internalization in comparison to their negatively or neutrally charged counterparts [86]. The diffusion of nanoparticles through cell walls and membranes is influenced by their size, with smaller nanoparticles potentially penetrating more deeply [87].

When nanoparticles enter plant cells, some signaling pathways may be activated [88]. The initial detection of nanoparticles that trigger subsequent signaling processes has been linked to recognition receptors on the cell surface, such as receptor-like kinases [89]. The complex web of interactions between nanoparticles and plant cells is highlighted by the activation of intracellular kinases and secondary messengers in response to nanoparticle uptake [90].

### 2.2. Cyanobacterial Intercellular Transportation

Cyanobacteria play a crucial role in the world’s biogeochemical cycles due to their capacity to harness light energy and transform carbon dioxide into organic molecules [91]. The complex mechanisms governing the physiology and relationships of cyanobacteria have been revealed thanks to the development of molecular and genomic technologies. However, the biological processes that control the intercellular communication and movement of cyanobacteria are still poorly understood [92].

Cyanobacteria have developed complex signal transduction and resource-sharing mechanisms to ensure coordinated responses to environmental signals. Small molecules, including nutrients and signaling chemicals, diffuse through channels known as septal junctions, one prominent method of intercellular transit. These septal connections, which are comparable to gap junctions in eukaryotic cells, enable direct cytoplasmic connections between neighboring cyanobacteria cells [93]. The function of outer membrane vesicles (OMVs) in facilitating the long-distance transit of bioactive chemicals and genetic material has also been clarified by recent investigations. OMVs, membrane-bound structures released from the cell surface, act as transporters for intracellular communication and may be crucial to the dynamics of the cyanobacterial community [94].

Cyanobacterial populations’ fitness and behaviors are significantly impacted by the interaction between intercellular transportation and cellular coordination [95]. Optimizing resource use and growth requires coordinated responses to environmental changes, such as those in nutrient availability and light intensity [96]. Intercellular movement makes it possible to share necessary metabolites such as fixed carbon and nitrogenous substances, which encourages population division [97]. Additionally, the transfer of genetic material via OMVs may promote horizontal gene transfer and genetic diversity, which may affect how well cyanobacteria can adapt to their environment. To fully understand the intricate dynamics of cyanobacterial communities in natural and manmade systems, it is imperative to comprehend these implications [98].

**Table 1:** Types and symbiotic forms of cyanobacteria symbionts.

Type	Moss Species and Relationship with Cyanobacteria	Cyanobacteria	Reference
Hornwort-cyanobacteria symbiont	*Anthoceros* sp. *Dendroceros* sp. *Notothylas* sp. *Phaeoceros* sp.	*Nostoc* sp.	[99,100,101,102,103]
Liverwort-cyanobacteria symbiont	*Anastrophyllum involutifolium* *Blasia* sp. *Cavicularia* sp *Chiloscyphusleptanthus* *Marchantia* sp. *Porella* sp.	*Nostoc* sp. *Calothrix* sp. *Stigonema* sp. *Chlorogloeopsis* sp.	[99,100,101,102,104]
Moss-cyanobacteria symbiont	*Acroschisma wilsonii* *Andreaea alpine* *Andreaea laxifolia* *Aulacomnium palustre* *Blepharidophyllum densifolium* *Bryum* sp. *Calliergon richardsonii* *Ceratodon purpureus* *Clasmatocolea humilis* *Cryptochila grandiflora* *Dendroligotrichum squamosum* *Dicranoloma chilense* *Ditrichum cylindricarpum* *Drepanocladus* sp *Drepanocladus exannulatus* *Dupontia* sp. *Grimmia* sp. *Heteroscyphus magellanicus* *Hylocomium splendens* *Paludella squarrosa* *Pleurozium schreberi* *Ptilium* sp. *Racomitrium Subcrispipilum* *Racomitriumlaevigatum* *Racomitrium lanuginosum* *Racomitrium didymium* *Sanionia uncinate* *Sphagnum lindebergii* *Sphagnum riparium* *Tomentypnum nitens* *Weisia controversa*	*Nostoc* sp. *Nostoc muscorum* *Calothrix* sp *Stigonema* sp *Scytonema* sp. *Anobena* sp. *Oscillatoria* sp. *Lyngbya* sp.	[99,100,101,102,105,106,107,108,109]

There are numerous biotechnological and environmental applications that could benefit from the knowledge learned from investigating cyanobacterial intercellular transfer. The effectiveness of bio-production processes such as biofuel synthesis and bioremediation could be improved by cyanobacterial consortia designed to exploit intercellular communication routes [110]. Additionally, using cyanobacteria’s inherent intercellular transportation systems should make it easier to transfer bioactive substances under controlled conditions for use in environmental engineering applications. Researchers can advance the development of sustainable technologies with broad-reaching benefits by taking advantage of the special characteristics of cyanobacterial communication networks. To fully understand the intricate dynamics of cyanobacterial communities in natural and manmade systems, it is imperative to comprehend these implications [111].

Metal nanoparticles (MNPs) are being used more often in a variety of industrial applications, which has resulted in a significant amount of them being released into the environment, raising worries about potential negative impacts on ecosystems and human health [112]. Due to their ability to absorb and detoxify heavy metals, plant-cyanobacteria systems for bioremediation have become more popular among the numerous methods to reduce the environmental effects of MNPs [113]. The kinetics of absorption, transport, and localization of nanoparticles can be studied using the interactions between MNPs and plant-cyanobacteria systems [113].

### 2.3. Root-Shoot Translocation

Water, minerals, hormones, and other vital substances travel in both directions through the root-shoot axis. Numerous physiological factors, including the availability of nutrients, hormone signaling, and climatic conditions, influence this transport, which takes place via complex systems involving vascular tissues such as xylem and phloem. According to recent studies, the selective transfer of chemicals between roots and shoots is governed by transport proteins, ion channels, and molecular gradients [114].

### 2.4. Systemic Distribution and Long-Distance Signaling

Plants communicate in a systemic manner that extends beyond the root shoot axis and involves the transmission of chemicals and information over long distances. The coordination of physiological responses, including defense systems, photosynthesis, and growth regulation, is fundamentally influenced by this process [115]. With its ability to support mass flow, the phloem is a crucial channel for systemic distribution, enabling the spread of pathogens, proteins, RNAs, and nutrients throughout plants. Auxin, cytokine, and abscise acid-based hormonal signaling, in particular, play a role in the coordination of systemic reactions [116].

### 2.5. Cyanobacterial Symbiosis

Because of their famed photosynthetic powers, cyanobacteria frequently create symbiotic partnerships with plants, aiding in nutrient uptake and stress tolerance. Complex molecular interactions are required for the development of cyanobacterial colonies in plant tissues, and specialized structures such as heterocysts and symbiotic interfaces facilitate nutrient exchange. Abiotic stress resistance, nitrogen fixation, and plant growth are all impacted by this mutually beneficial connection [117].

## 3. Molecular Insights into Nanoparticle Toxicity

**Figure 2:**
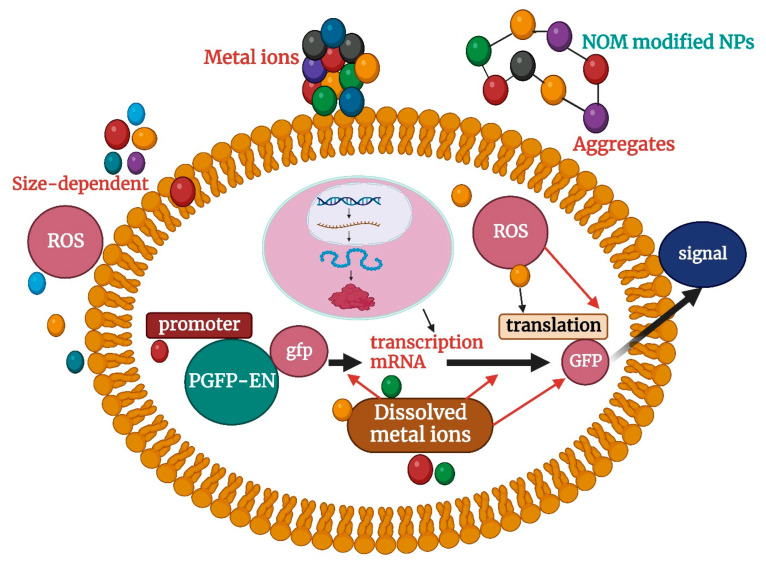
Toxicological effects of nanoparticles on *Escherichia coli*.

Beginning with cellular uptake, which is controlled by NP size, shape, surface charge, and composition, interactions between NPs and biological systems begin. These physicochemical characteristics have an important role in determining cellular internalization mechanisms, including endocytosis and direct penetration, according to recent studies. Electrostatic interactions may be important because they show that positively charged silver nanoparticles have increased cellular absorption compared with their negatively charged counterparts [118]. Numerous investigations have documented the genome toxicity caused by NP, which includes mutations, chromosomal abnormalities, and DNA strand breaks. These consequences may result from ROS-related secondary effects or from physical damage caused by direct interactions between NPs and DNA. It was revealed that the production of ROS and subsequent activation of DNA repair pathways by gold nanoparticles could cause DNA damage [119]. The key methods for toxicological appraisal of nanoparticles, as described in the search results, include a combination of in vitro and in vivo approaches. In vitro studies using cell lines are crucial for assessing the acute damaging effects of nanoparticles in specific cellular environments [120,121]. Genotoxicity testing is also used to evaluate the potential of nanoparticles to cause genetic damage such as mutations or chromosomal aberrations. Calculating the No Observed Adverse Effect Level (NOAEL) is an important step in nanoparticle toxicity testing, which helps determine the safe dose levels for further studies [122,123]. However, extrapolation of cell-based data into in vivo scenarios is problematic due to the extreme conditions in vitro, so in vivo testing remains crucial for assessing biodistribution, bioaccumulation, and systemic toxicity [124]. To fully investigate the toxicity and potential risk of a particular nanomaterial, a combination of multiple in vitro tests is required. Table 2 lists the toxicity of several typical nanoparticles that have been evaluated using different biological assessment models. The toxicity of nanoparticles to organisms mainly includes the following four aspects: (1) nanoparticles with nanoscale are easy to cross the cell membrane into the cell, easy to damage the cell membrane and interfere with the physiological activity in the cell; (2) Nanoparticles have high activity, and the generated reactive oxygen species (ROS) on the one hand is easy to damage the cell membrane, destroy the permeability of the cell, hinder the exchange of materials between the cell and the outside world, and easily cause protein denaturation; On the other hand, ROS produced by nanoparticles can stimulate the oxidative stress pathway in cells, resulting in cell damage [2]; (3) Nano-oxygen is applied to some nanoparticles that can dissolve metal ions (such as programmed copper, cadmium selenide, etc.), and the dissolved metal ions also enhance the toxicity of nanoparticles in terms of nanoscale. Taking the study on the toxicity of particles to particles in Figure 2 as an example, the toxicity of *Escherichia coli* nanoparticles can be divided into the following aspects: First, the nanoparticles have a large specific surface area, which can fully interact with the cell membrane of *E.coli* and destroy the permeability of cells, thus causing cytotoxicity; Secondly, ROS produced by nanoparticles themselves and possibly dissolved metal ions can cause toxicity in *E. coli*. Moreover, nanoparticles entering cells can stimulate oxidative pathways in cells, resulting in oxidative stress behavior, interfering with protein expression in cells, or post-transcriptional translation [113]. Finally, nanoparticles entering the cell can interact with proteins and their DNA in the cell, causing them to lose certain functions [124]. In previous toxicity studies, a single biological model was typically used to assess or study the toxicity of nanoparticles. Commonly used model organisms have included bacteria [122], algae [125], fish [126], zooplankton [127], mammalian cell lines [128], and so on. Studies have shown that nanoscale particles can seriously impair the physiological activities of cells, subcells, and proteins and even lead to cell death. The main hazards include entry and deposition in the relevant cells (nerve cells, hepatocytes, etc.) [128], protein degeneration and reduced enzyme activity [129], and genotoxicity [130]. DNA mutation and cell membrane damage [131] Oxidative stress [124] and mitochondrial damage [127] affect the expression of functional proteins [132] and immunity [133].

ROS represents engineering (such as hydroxyl radical •OH, active oxygen singlet oxygen O_2_^−^, etc.); NOM stands for natural organic matter; NPs stands for nanoparticle.

Surface changes, antioxidant co-administration, and tailored medication delivery have all been investigated as methods to reduce NP toxicity. Surface customization of NPs with biocompatible ligands or polymers can alter their interaction with cells and lessen toxicity. It has been demonstrated that NP-induced oxidative stress can be reduced by the co-administration of antioxidants such as vitamin C or N-acetyl cysteine. Additionally, the development of NPs for targeted drug delivery offers hope for reducing side effects while enhancing therapeutic efficacy [164].

### 3.1. Plants and Cyanobacteria Cellular Reactions to Exposure to Metal Nanoparticles

Electronics, healthcare, and agriculture have undergone radical change due to the rapid development and commercialization of metal nanoparticles (NPs). However, given the frequency with which NPs are used, there are worries regarding possible biological and environmental effects. Due to their crucial ecological roles, plants and cyanobacteria in particular have drawn a lot of interest to the interactions between metal NPs and living organisms. Plants are the principal producers that support terrestrial ecosystems by affecting carbon sequestration, soil structure, and nutrient cycling [165].

As the most common photoautotrophic microorganisms in aquatic environments, cyanobacteria make a considerable contribution to the world’s nitrogen and carbon cycles. Understanding the effects of metal NPs on plants and cyanobacteria is essential for predicting and minimizing possible disruptions to ecosystem function, as metal NPs can easily enter terrestrial and aquatic systems [166].

Silver (Ag), gold (Au), zinc oxide (ZnO), and iron oxide (Fe_3_O_4_) are only a few examples of the wide variety of materials that make up metal NPs, each with unique physicochemical characteristics. Plants and cyanobacteria use various methods, including NP size, surface charge, and environmental factors, to absorb metal NPs. NPs have the ability to cross cell membranes and walls in plants to access intracellular compartments. Because of their straightforward cell structure, cyanobacteria can directly absorb NPs by passive diffusion or active transport [167].

### 3.2. Oxidative Stress and Its Implications for Plant-Cyanobacteria Health

In the fields of plant biology and microbial ecology, oxidative stress, defined as the excessive accumulation of ROS beyond the cellular antioxidant capacity, has attracted a lot of attention [168]. Because they are sessile organisms, plants are regularly exposed to a variety of environmental stresses, from biotic variables like herbivores, diseases, and symbiotic bacteria to abiotic factors such as light intensity, temperature changes, and nutrition availability. Cyanobacteria, which can perform photosynthesis and nitrogen fixation, are among these symbiotic partners and create complex relationships with numerous plant species throughout varied habitats [169].

Superoxide anion (O_2_), hydrogen peroxide (H_2_O_2_), and hydroxyl radicals (•OH) are three examples of ROS that are produced naturally during aerobic metabolism and photosynthesis [170]. ROS are crucial signaling molecules in low quantities, but when they build up excessively, they can cause cellular damage and oxidative stress. To maintain redox equilibrium and fight oxidative stress, plants and cyanobacteria have developed complex defense systems that include enzymatic antioxidants (such as superoxide dismutase, catalase, and peroxidases) and non-enzymatic antioxidants (such as ascorbate, glutathione). Nevertheless, a variety of external stressors can upset the equilibrium between ROS generation and scavenging, leading to oxidative stress-related cellular damage [171].

### 3.3. Signaling Pathways Triggered by Nanoparticle-Induced Stress in Mutualistic Systems

In species from numerous taxonomic groups, exposure to nanoparticles can impair redox balance, disturb cellular homeostasis, and induce a variety of stress reactions [172]. These stressors then trigger a complicated network of signaling channels that control defense and adaptation systems. Mutualistic partners can communicate and control their physiological reactions in the face of stress caused by NP thanks to the complex crosstalk between these pathways [173]. The essential elements of the complex signaling networks triggered in mutualistic organisms under NP stress include reactive oxygen species (ROS) signaling, hormone-mediated pathways, and phytochemical cascades. These signaling pathways help to shape the overall outcome of reciprocol interactions in the presence of NPs, in addition to mediating partner-specific responses [174].

## 4. Enhancement of Nutrient Uptake and Availability

Growth, development, and production are crucially influenced by how effectively cyanobacteria and plants acquire and use vital nutrients. However, the availability of nutrients, particularly micronutrients, in soil and aquatic habitats is often limited, leading to suboptimal physiological conditions and lower yields [175]. Metal nanoparticles (MNPs) have drawn a lot of interest due to their distinct physicochemical characteristics and prospective uses in biotechnology and agriculture. In order to overcome the problems caused by nutrient deficiency and improve the nutritional status of plants and cyanobacteria, exploiting the ability of MNPs to improve nutrient uptake and availability has great potential [113].

### 4.1. MNPs as Carriers of Essential Nutrients: Root Interactions and Cyanobacterial Associations

Due to their high surface-to-volume ratio and nanoscale size, MNPs have improved reactivity and solubility, making them efficient transporters of vital nutrients [176]. Root interactions are important for nutrient uptake, and MNPs can alter rhizosphere conditions to change the solubility and availability of nutrients. In addition, cyanobacteria can create complex relationships with plants because they are skilled nitrogen fixers and extracellular polymer makers. The addition of MNPs to these connections has the potential to increase nutrient transfer and build cooperative networks of nutrient exchange, improving nutrient flow and bioavailability [177].

### 4.2. Nutrient-Use Efficiency and Crop Yield Enhancement through Plant-Cyanobacteria Synergy

To ensure sustainable crop production and reduce environmental consequences, modern agriculture must prioritize optimizing nutrient use efficiency [178]. A promising method for reaching this goal is the symbiotic relationship between plants and cyanobacteria. MNPs can increase nutrient delivery and uptake, which can intensify the advantages of plant-cyanobacteria synergy. MNPs are carriers of both critical nutrients and bioactive chemicals. This interaction not only promotes efficient nutrient utilization but also increases crop production and quality, addressing the issue of global food security [179].

### 4.3. Ecological and Agricultural Implications

MNP inclusion in nutrient management plans has significant ecological and agricultural effects. MNPs can be used as precise instruments in agroecosystems to address certain nutrient deficiencies and reduce the overuse of traditional fertilizers [180]. In addition, the use of MNPs in ecologically delicate locations can encourage nutrient cycling that is sustainable, lowering the risk of eutrophication and nutrient runoff. However, the ecological effects of MNPs need to be carefully considered, and their long-term effects on soil health, microbial populations, and ecosystem dynamics must be evaluated holistically [181].

## 5. Stress Tolerance and Disease Resistance

Beyond symbiotic interactions, the complicated dance between plants and cyanobacteria includes mechanisms that allow these organisms to endure stress and fend off pathogenic threats. Partnerships between plants and cyanobacteria have drawn interest because of their ability to ameliorate adverse environmental conditions and increase crop productivity [182]. Agricultural systems face severe challenges from stressors, including salt and drought, demanding creative strategies to increase stress tolerance. Furthermore, the necessity of strong defense mechanisms is highlighted by the sensitivity of plants and cyanobacteria to pathogenic agents [183].

### 5.1. Nanoparticle-Mediated Stress Responses

Recent studies have shown how nanoparticles can be used to control stress responses in plant-cyanobacteria systems. Due to their distinctive physicochemical characteristics, nanoparticles have been demonstrated to have an impact on a number of biological functions, including stress-related signaling pathways [184]. By boosting antioxidant defense mechanisms and osmoprotectant accumulation, nanoparticle therapies have been suggested as viable ways to improve stress tolerance. Notably, nanoparticles have been linked to the control of gene expression networks associated with stress responses, thereby strengthening stress tolerance mechanisms [185].

### 5.2. Reinforcement of Pathogen Defense Mechanisms

Pathogens present considerable obstacles to the interactions between plants and cyanobacteria, in addition to abiotic stresses. Different diseases have the potential to upset the delicate balance of these alliances, which would limit plant development and damage cyanobacterial activity [186]. According to recent research, nanoparticle treatments may cause both partners to produce antimicrobial chemicals and upregulate defense-related genes. By priming defense mechanisms through the use of nanoparticles, disease resistance is increased, preserving the stability of plant-cyanobacterial systems [187].

### 5.3. Mitigation of Abiotic Stress Factors

The adaptability and durability of plant-cyanobacteria systems are demonstrated by their versatility in reducing abiotic stress effects. In many different agricultural environments, salt and drought are common stressors that can seriously hinder development and productivity. By improving water retention and nutrient uptake, nanoparticle therapies have been demonstrated to lessen the negative impacts of abiotic stresses and maintain cellular homeostasis in both plants and cyanobacteria. The potential of nanoparticle interventions to ensure the stability and productivity of plant-cyanobacteria interactions is highlighted by a multimodal approach to stress mitigation [188].

## 6. Impact on Key Plant and Cyanobacterial Processes

By facilitating energy flow and nutrient cycling, plants and cyanobacteria, as primary producers, play critical roles in ecosystem functioning [189]. However, because their performance is closely linked to current environmental circumstances, they are vulnerable to several stresses. The development, metabolism, and reproduction of plants and cyanobacteria are significantly impacted by environmental stressors such as high temperatures, pollution, and changed nutrient availability [190].

### 6.1. Impact on Photosynthesis and Carbon Fixation

In both plants and cyanobacteria, photosynthesis is essential for obtaining energy and assimilating carbon. However, stress-related modifications to the photosynthetic apparatus, such as changes in pigment composition and electron transport, can seriously impair the efficiency of energy conversion [191]. Additionally, the complex regulatory networks that control carbon fixation are prone to disruption under stress, leading to imbalances in the distribution of fixed carbon to crucial biomolecules [192].

The development and metabolic processes of plants and cyanobacteria are significantly influenced by the availability of critical nutrients. Elements can be out of balance due to stress-induced changes in nutrition absorption systems and assimilation pathways, endangering cellular homeostasis and overall fitness [193].

### 6.2. Effects of Nanoparticles on Photosynthesis, Energy Conversion, and Nitrogen Fixation by Cyanobacteria

The process of photosynthesis, which transforms light energy into chemical energy, is a crucial biological phenomenon that powers the ecosystems on Earth. In order to increase the efficiency of photosynthetic production in cyanobacteria, nanoparticles have been studied as potential modulators of light absorption and energy transfer. It is possible to improve light-harvesting complexes and fine-tune photosynthetic pigments through the controlled transport of nanoparticles to cyanobacterial cells, which will ultimately increase energy absorption and conversion [194].

Cyanobacteria have a surprising variety of metabolic pathways, including the storage and conversion of energy. Through better electron transport chains and improved synthesis of energy-rich compounds, it was investigated whether nanoparticles can support bioenergy production. Furthermore, nanoparticle-induced changes in cellular redox balance and electron flow have the ability to redirect metabolic flows toward desired end products [91].

### 6.3. Effects on Root Architecture, Mycorrhizal Associations, and Cyanobacterial Aggregates

Key elements of terrestrial ecosystems include root architecture, mycorrhizal connections, and cyanobacterial aggregates, which affect nutrient intake, soil structure, and plant health. Understanding these variables’ complex relationships is crucial for ensuring the resilience and sustainability of ecosystems. The spatial arrangement of roots in the soil is influenced by root architecture, which also affects nutrient and water uptake [195]. Root growth patterns are influenced by environmental factors such as gravity, moisture, and nutrient availability. A growing body of evidence points to the crucial role that microorganisms, such as mycorrhizal fungi and cyanobacteria, play in directing root branching, elongation, and tip growth [196].

**Figure 3:**
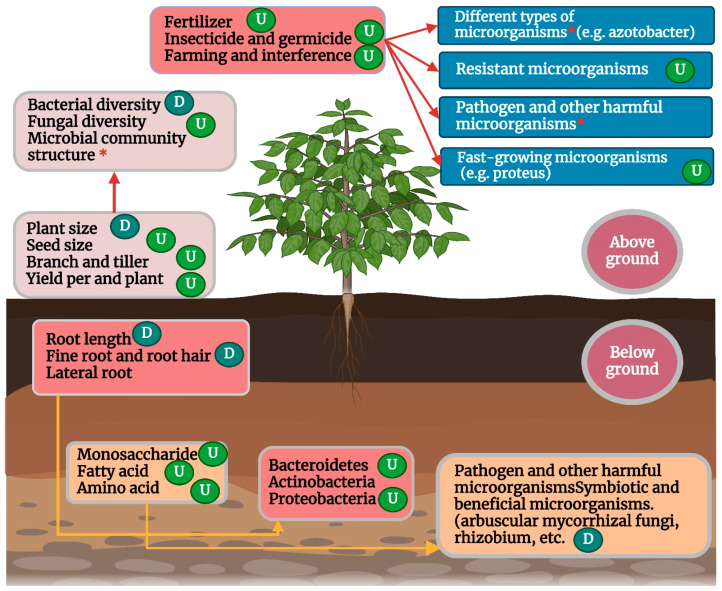
Effects of domestication on microbiome community composition and diversity of crops (U) indicate significant increase and (D) decrease, respectively; (*) indicates a significant difference.

The process of domestication has significantly altered crop root configuration, including, but not limited to, root branching, length, biomass, volume, anatomical structure, and surface area [197], which influence the recruitment and colonization of root microbial communities in wild and modern crop varieties [198] (Figure 3). Under modern intensive farmland management with high density and high nutrient input, crop domestication usually leads to fewer fine roots, shallower roots, increased lateral roots, and a lower root-to-stem ratio of crops [199,200] and this can have a profound impact on root microbes. For example, [201] showed that fine roots (or root hairs) of plants enrich more diverse microbial communities than coarse roots, and the abundance of root microorganisms in fine roots is generally higher than that in coarse roots. The authors of [202] also found that fine roots were more likely to promote microbial growth, while [203] found that the absence of root hair significantly reduced the complexity of rhizosphere microbial communities. The authors of [204] found that the root system of domesticated modern kidney beans was “coarser” than that of wild kidney beans, resulting in a significantly lower relative abundance of Bacteroides in the rhizosphere microorganisms of modern kidney beans. Therefore, the reduction of fine roots caused by domestication may be an important reason why the diversity of root bacteria and fungi of most modern crop varieties is lower than that of their wild ancestors.

## 7. Phytoremediation Potential

Anthropogenic environmental contamination has become a global issue, calling for creative and long-lasting restoration techniques. An engineering method called phytoremediation uses the inherent capacities of plants and the microbes that live with them to mitigate various pollutants. This paper offers a thorough summary of the potential, methods, applications, difficulties, and recent developments in phytoremediation. Rhizofiltration, phytostabilization, phytoextraction, and phytovolatilization are only a few of the many mechanisms involved in phytoremediation. Through their root systems, plants can absorb, change, immobilize, or destroy contaminants, thereby cleansing the environment. Numerous studies have shown how these processes work in various plant species and polluted environments [205].

Plant-microbe interactions are essential to the efficacy of phytoremediation. The rhizosphere’s microorganisms support pollutant transformation and nutrient uptake, improving plant health and pollutant degradation. Recent research has emphasized the value of rhizobacterial populations and mycorrhizal connections in boosting phytoremediation effectiveness [206].

The efficacy of phytoremediation has improved thanks to developments in genetic engineering. Increased pollutant uptake, tolerance, and degradation rates may be due to a genetic change. The development of hyperaccumulator plants specifically suited to environmental remediation is possible thanks to advances in synthetic biology. Plants that overexpress metal transporters and enzymes involved in pollution metabolism have been successfully created by researchers [207].

### 7.1. Mechanisms of Phytoremediation Assisted by Metal Nanoparticles

Metal nanoparticles interact with plants and their rhizosphere through a variety of methods, including increased adsorption surface area, enhanced metal uptake through root penetration, and accelerated translocation in plant tissue. These interactions often result in increased plant development, reduced metal toxicity, and improved metal sequestration. One of the main factors affecting the effectiveness of nanoparticles is how they affect metal speciation and availability in soil [208].

### 7.2. Synergistic Effects on Plant-Microbe Interactions

In addition to plant physiology, metal nanoparticles also influence the composition and activity of rhizospheric microbial communities. These adaptations can lead to increased metal mobilization and transformation, increased plant stress tolerance, and increased nutrient availability. The potential for nanoparticle-assisted phytoremediation to increase the overall effectiveness of pollutant removal is highlighted by the complex interplay between nanoparticles, plants, and microorganisms [209].

Numerous metal-contaminated areas have been successfully remediated using metal nanoparticle-assisted phytoremediation. Numerous metal nanoparticles, including zero-valent iron, iron oxide, and titanium dioxide, have been the subject of case studies that have demonstrated their efficacy in accelerating the buildup and removal of metals from contaminated soils and streams. These studies give important information on how this strategy can be used in practice [210].

### 7.3. Ecological Considerations, Cyanobacterial Contributions, and Long-Term Effectiveness

Despite the positive results, there are still issues that need to be resolved, including the stability of nanoparticles, possible ecotoxicological effects, and regulatory issues. The fat and behavior of nanoparticles in the environment, as well as their long-term implications for ecosystems, are still being researched. The development of sustainable nanoparticle-assisted phytoremediation solutions must take into account the optimization of nanoparticle synthesis technologies and the assessment of their environmental effects [211].

## 8. Cutting-Edge Developments in Agricultural Nanotechnology

Population growth, climate change, and a decline in arable land pose growing problems for agriculture. By exploiting the special properties of nanoparticles to improve plant growth, nutrient supply, and pest control, agricultural nanotechnology offers promising answers. Nanoparticles are receiving attention because of their potential to improve soil structure, nutrient retention, and water availability. Examples include nanoclays, metal nanoparticles, and carbon-based nanomaterials [212]. The potential of nanomaterials to increase crop production and stress tolerance has been demonstrated. Man-made nanoparticles such as silicon and zinc oxide nanoparticles have been shown to improve plant growth, photosynthesis, and nutrient absorption [213]. Agrochemical nanoformulations such as insecticides and fertilizers enable controlled release and targeted delivery, reducing the amount of chemicals required and minimizing environmental impact [214]. By providing real-time information about soil moisture, nutrient levels, and pest infestation, nanotechnology-enabled sensors play a critical role in precision agriculture. These sensors, often combined with data analytics and wireless networks, help farmers make informed decisions, maximize resource utilization, and reduce input waste [215]. Furthermore, nanoscale biosensors are being developed to quickly detect plant diseases and ensure immediate intervention to reduce crop losses [216].

### 8.1. Integrating Cyanobacterial Solutions

Due to their ability to accumulate lipids and carbohydrates, cyanobacteria are potential candidates for biofuel production. The main objectives of the research were to improve pathways for lipid production and increase biomass productivity through genetic engineering and cultivation techniques. Due to improvements in metabolic engineering, strains of cyanobacteria capable of producing biofuels such as biodiesel and bioethanol have been created [217]. It is important to highlight the role that cyanobacteria play in reducing carbon dioxide levels and helping to adapt to climate change. Although cyanobacteria blooms are often considered destructive, they have the ability to store carbon. Cyanobacteria provide a natural solution for carbon capture and storage by absorbing atmospheric carbon and incorporating it into their biomass [218]. The use of cyanobacteria in environmentally friendly treatment methods is increasing in popularity due to their ability to absorb nutrients and toxins from wastewater. Photobioreactors and constructed wetlands are two examples of cyanobacteria-based wastewater treatment technologies that provide an environmentally friendly method of nutrient removal and water filtration. These systems produce biomass that, in addition to wastewater treatment, is also suitable for the production of bioenergy [219].

### 8.2. Cyanobacterial Biofertilizers and Soil Fertility

Cyanobacteria are essential for soil nutrient enrichment and the nitrogen cycle. Improved soil fertility and plant development are the result of their capacity to fix atmospheric nitrogen and secrete chemicals that encourage growth. According to research, using cyanobacterial biofertilizers can increase agricultural yields, especially in soils with low nitrogen levels. Additionally, the interactions between cyanobacteria and soil microbes can create a favorable rhizosphere environment, enhancing nutrient availability and plant health [220]. The overuse of artificial fertilizers has sparked worries about resource depletion and environmental degradation. A sustainable solution is provided by cyanobacterial biofertilizers, which increase the effectiveness of nutrient use and decrease the need for synthetic fertilizers. Studies have shown that cyanobacterial biofertilizers can improve nutrient uptake, maximize fertilizer use, and reduce nutrient leaching, thereby reducing the adverse impacts of nutrient runoff on the environment [221]. Precision farming methods allow for site-specific management of agricultural inputs thanks to developments in remote sensing, geographic information systems (GIS), and data analytics. By combining cyanobacterial biofertilizers with precision farming, it is possible to apply them in a way that is specifically matched to the nutrient needs of various crop zones. This strategy maximizes the advantages of using biofertilizers while optimizing nutrient distribution, cutting waste, and reducing waste [222]. It has been demonstrated that cyanobacterial biofertilizers increase crop tolerance to a variety of abiotic conditions, including salt, drought, and heavy metal toxicity. Along with greater nutritional availability, the growth-promoting compounds released by cyanobacteria help plants develop stronger stress resistance systems. Combining precision farming methods with the use of biofertilizers can help identify areas that are more vulnerable to stress and perform tailored interventions to lessen its effects [223].

### 8.3. Role of Cyanobacteria in Nanoparticle Applications

Cyanobacteria have the innate ability to synthesize nanoparticles by reducing metal ions. A specific method for regulating the size, shape, and composition of nanoparticles is enabled by their capacity to metabolize a variety of substrates and modulate biological functions. Recent studies have shown that cyanobacteria are capable of biosynthesizing metallic, metal oxide, and semiconductor nanoparticles, which have uses in photonics, catalysis, and other fields [224].

Nanoparticles must have their surfaces functionalized in order to improve their stability, biocompatibility, and specific uses. Through the use of genetic engineering or environmental modification, cyanobacteria provide a biogenic substrate for the synthesis of nanoparticles with customized surface properties. This provides opportunities for the development of bio-inspired, biocompatible nanoparticles with improved capabilities for imaging in medicine, drug delivery, and environmental sensing [225].

### 8.4. Ethical Considerations and Regulatory Implications

The use of cyanobacteria for the synthesis of nanoparticles raises ethical questions about the release of contaminants into the environment, unforeseen consequences, and potential hazards to ecosystems and human health. Rigorous risk assessment, transparency, and adherence to regulatory frameworks are necessary to strike a balance between technological growth and responsible innovation. Consideration must also be given to the moral ramifications of patenting biogenic nanoparticles produced by natural processes. Collaborations between scientists, politicians, ethicists, and stakeholders across other disciplines are necessary to strike a balance between the advantages of cyanobacteria-mediated nanoparticle applications and ethical issues. The development of open guidelines, encouraging public participation, and advancing sustainable innovation through well-informed decision-making should be the main goals of responsible research and innovation [226].

## 9. Current Trends in Agricultural Nanotechnology

The need to enhance food production to meet the demands of an expanding global population while reducing environmental effects is one of the many difficulties facing agriculture. By offering methods to improve agricultural processes at the nanoscale, nanotechnology offers creative responses to these problems. Crop yields, resource use, and overall agricultural sustainability can be improved by nanoscale manipulation of materials and structures [227].

The development and use of nanoparticles for crop improvement are two major trends in agricultural nanotechnology. The potential for improving nutrient uptake, enhancing plant growth, and reducing stress factors has been demonstrated by nanoparticles such as nanopesticides and nanofertilizers. By directly delivering nutrients to plant cells, these nanoparticles can improve nutrient uptake efficiency while lowering environmental contamination [228].

**Figure 4:**
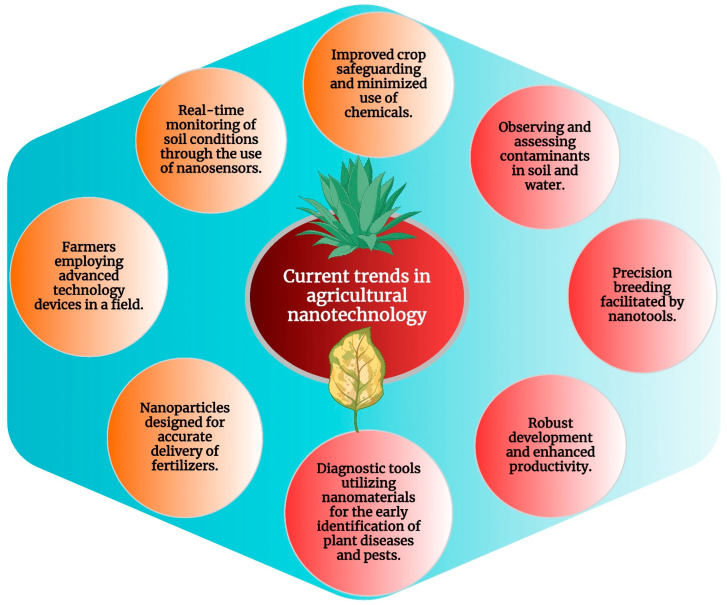
Current trends in agricultural nanotechnology.

By giving soil managers access to real-time information on soil moisture, nutrient levels, and microbial activity, nanoscale sensors play a crucial role in soil management. These nanosensors provide farmers with exact information to optimize irrigation and fertilization practices, thereby improving resource management and increasing crop output. Additionally, nanoparticles can be employed to enhance soil structure and water retention, promoting long-term sustainability and soil health [229]. Several recent studies have demonstrated the significant potential of nanotechnology in enhancing crop productivity, resource utilization, and environmental impacts in the context of precision and sustainable agriculture. These studies have explored various nanotechnological interventions, such as foliar and soil bio-augmentation, to address challenges in agriculture, including climate change, soil contamination, and increasing food demands. The application of nanotechnology in agriculture is increasingly recognized as a critical aspect of precision farming, offering new solutions to combat existing problems and improve modern farming practices. Figure 4 provides a diagrammatic representation of the application of nanotechnology in precise and sustainable agriculture and current trends in the field. The interaction between plants and nanomaterials is the focus of these studies and highlights the potential for developing novel and desirable materials for crop improvement. While the use of nanotechnology in agriculture holds promise for sustainable green agriculture, it also presents challenges such as nanotoxicity and environmental impact, which require careful regulation and long-term evaluation.

### 9.1. Integrating Metal Nanoparticles into Modern Agricultural Practices

It has been demonstrated that adding metal nanoparticles, such as copper, gold, and silver, can improve plant growth, disease resistance, and nutrient uptake. The antibacterial capabilities of metal nanoparticles can reduce plant diseases and enhance crop health [230]. They have shown improved nutrient efficiency and decreased chemical discharge in nanopesticides and nanofertilizers, which support sustainable agriculture practices [231]. Additionally, the sustained effects of the controlled release of metal nanoparticles from nanomaterials eliminate the need for repeated applications. Modern agricultural techniques that incorporate metal nanoparticles have a significant promise for increasing yields while reducing their negative environmental effects [232].

### 9.2. Integrating Cyanobacterial Solutions

Photosynthetic microorganisms, called cyanobacteria, provide a sustainable solution to agricultural problems. Opportunities for biofertilization and promoting plant growth are provided by their capacity to fix atmospheric nitrogen and produce bioactive chemicals [233]. It has been shown that cyanobacterial inoculants increase soil fertility, nutrient availability, and water retention [220]. Additionally, cyanobacteria support soil health and microbial diversity by enhancing soil structure through the production of exopolymeric compounds. A natural and environmentally benign method of increasing agricultural output while lowering chemical inputs is the incorporation of cyanobacterial solutions [234].

### 9.3. Synergies with Precision Farming, Sustainable Agriculture, and Cyanobacterial Biofertilizers

The combination of cyanobacterial biofertilizers, precision farming, and agricultural nanotechnology has enormous promise for maximizing resource usage and reducing environmental impact. Utilizing real-time data, precision farming methods such as remote sensing and GPS-guided machinery allow for the targeted application of nanomaterials and cyanobacterial solutions [235]. This interaction improves crop quality, lowers waste, and improves fertilizer management. Additionally, the combination of cyanobacterial biofertilizers and precision farming promotes long-term agricultural sustainability by improving the health and fertility of the soil. The joint application of these technologies represents a major step towards a future where agriculture is more effective and environmentally sensitive [236].

### 9.4. Balancing Benefits, Ethical Concerns, and the Pivotal Role of Cyanobacteria in Nanoparticle Applications

It is quite possible to maximize resource use and reduce environmental effects by combining agricultural nanotechnology, precise farming, and cyanobacterial biofertilizers. The focused application of nanomaterials and cyanobacterial solutions is enabled by precision farming techniques, such as remote sensing and GPS-controlled devices, based on real-time data [237]. This interaction optimizes fertilizer management, lowers waste, and raises crop quality. Additionally, the use of biofertilizers derived from cyanobacteria in conjunction with precision farming promotes long-term soil fertility and health. A substantial step towards a more effective and environmentally responsible agricultural future has been made by the coordinated use of these technologies [238].

## 10. Future Prospects and Challenges

### 10.1. Unexplored Areas of Research in Plant-Cyanobacteria-Nanoparticle Interactions

Although tremendous progress has been made in understanding the interactions between cyanobacteria and plants, little is known about how nanoparticles may affect this complex relationship. Recent studies demonstrate that nanoparticles and cyanobacterial bioinoculants may work in concert to improve plant growth, stress tolerance, and nutrient uptake. However, further research is needed to determine the underlying mechanisms and precise effects of nanoparticles on cyanobacterial symbiosis and subsequent plant responses. Opportunities exist to decipher innovative mechanisms that may support sustainable agriculture and environmental health in this unexplored zone [239].

### 10.2. Regulatory Aspects, Ethical Considerations, and the Potential of Cyanobacterial Bioinoculants

The ability of cyanobacterial bioinoculants to fix atmospheric nitrogen, improve soil fertility, and stimulate plant growth highlights their potential to revolutionize sustainable agriculture. The use of bioinoculants, such as cyanobacteria, is subject to growing regulatory frameworks that seek to assure product efficacy, safety, and minimal environmental impact. Biosafety, intellectual property rights, and the equal sharing of rewards are all ethical issues. It is crucial to strike a balance between innovation and responsible use. By lowering the use of chemical inputs, preserving resources, and enhancing soil health, cyanobacterial bioinoculants have the potential to revolutionize agricultural practices; however, their integration must be achieved within strict regulatory and ethical frameworks [239,240].

### 10.3. Steps toward Responsible and Safe Utilization of Nanoparticles in a Cyanobacteria-Augmented Environment

A cautious approach is required when the agricultural world investigates the incorporation of nanoparticles into systems enhanced with cyanobacteria. To examine potential nontoxicity, effects on unintended organisms, and long-term repercussions on soil and aquatic ecosystems, thorough risk assessments must be carried out. Unintended consequences can be reduced by including life cycle analyses and creating standardized methods for nanoparticle formulation and use. To create policies that encourage the responsible use of nanoparticles in cyanobacteria-augmented environments while minimizing ecological and human health hazards, collaboration between scientists, policymakers, and stakeholders is crucial [147].

## 11. Conclusions

In the field of agricultural science, the combination of nitrogen-fixing cyanobacteria and nanoparticle-based methods holds great promise for improving crop yields. This study explores the detailed molecular processes that make this partnership effective and how it can be used in farming. Nitrogen-fixing cyanobacteria have evolved special traits that help them deal with low levels of nitrogen in the soil. Meanwhile, nanoparticles provide new ways to deliver nutrients to plants and make them stronger. By closely studying how these two work together, from the tiniest levels of molecules to larger ecosystems, we learn more about how they can boost crop growth and output. In the forthcoming stages, the amalgamation of cyanobacteria and nanoparticles demonstrates promise in fostering sustainable agricultural methodologies, reducing dependency on synthetic fertilizers, and mitigating environmental impacts. Nevertheless, comprehensive scrutiny of the ecological and ethical ramifications of these methodologies is indispensable to guarantee prudent implementation and stewardship of agricultural resources. To conclude, the amalgamation of cyanobacteria and nanoparticles unveils an enticing frontier for agricultural progression, providing avenues for heightened efficiency and ecological sustainability in food production. Sustained research efforts and thoughtful consideration are essential as we traverse the dynamic terrain of agricultural innovation.

## Figures and Tables

**Table 2:** Toxicity studies of typical nanoparticles based on different biological evaluation models.

Kinds of NPs	Tested Objects	Toxicological Effects	References
nanoparticle containing carbon	human lung epithelial cells A549	inhibition 22% (40 g/cm^2^ exposure 18 h); 15% DNA damage (40 µg/cm^2^ exposure 4 h)	[134]
	alveolar macrophage	(single-walled) inhibition 20% (1.41 µg/cm^2′^ exposure 6 h), (multi-walled) inhibition 14% (22 µg/cm^2^ exposure 6 h)	[135]
	human epidermal keratinocytes (HEKs)	(multi-walled) altered the expression of 36 proteins (exposure 24 h): altered 106 (exposure 48 h)	[136]
	Cupriavidus metallidurans CH34	CNT are internalized (10 µg/mL exposure 24 h)	[137]
	Escherichia coli MG1655	CNT are internalized (10 µg/mL exposure 24 h)	[137]
	Drosophila melanogaster	strongly adhering CB significantly reduced survivorship	[138]
	adult oysters	40% cell damage (100 µg/L, exposure 4 days)	[139]
	*Crassostrea virginica*	embryonic development and lysosomal destabilization (10 µg/L exposure 24 h)	[140]
	Daphnia: fathead minnow	THF-*nC_go_*-exposed fish 100% mortality (6 and 18 h), water-46 stirred-*nC_60_*-exposed fish no effects (48 h). However significantly increased expression of CYP2 family isozymes in liver	[125]
	*Escherichia coli*	attributed to photocatalytically generated ROS. Exerts ROS-independent oxidative stress (10 mg/L exposure 15 min)	[140]
metal oxide	*Drosophila melanogaster*	less adhering was indistinguishable from unexposed control	[141]
	human lung epithelial cells A549	DNA damaged 19% (40 µg/cm^2^ exposure 4 h);	[134]
	*Daphnia magna*	19/25 acute toxicity EC_50_ > 100 mg/L (exposure 48 h)	[142]
	*Chlamydomonas reinhardtii*	transient up-regulation of genes as low as 1.0 mg/L: *Sodl.gpx*, *cat* and *ptox2* (exposure 6 h);	[137]
	*Caenorhabditis elegans*	LC_50_ 80 mg/L (exposure 24 h)	[138]
	*Cupriavidus metallidurans* CH34	cell internalized and induced significant ROS production at 500 mg/L;	[137]
	*Escherichia coli* MG1655	cell internalized and induced significant ROS production at 500 mg/L;	[137]
	brain microglia (BV2)	produced ROS; neurotoxicity (2.5–120 mg/L exposure 1, 6, 18 h)	[127]
	*Bacillus subtilis*: *Escherichia coli*	low toxicity on the three tested bacteria (20 mg/L exposure 24 h	[140]
	*Pseudomonas fluorescens*	inhibition ratio 38% (40 g/cm^2^ exposure 18 h), DNA damaged 12% (exposure 4 h)	[134]
	human lung epithelial cells A549	LC_50_ (2.3 mg/L exposure 4 h)	[143]
	*Caenorhabditis elegans* RAW 264.7 and BEAS-2B	generated ROS, inflammation, cell death (25 pg/mL exposure1–16 h)	[144]
	*Daphnia magna*	acute toxicity; EC_50_ = lmg/L (exposure 48 h)	[144]
	*Caenorhabditis elegans*	LC_50_ 2.3 mg/L (exposure 24 h)	[145]
	*Escherichia coli*; *Staphylococcus aureus*	inhibition concentrations > 3.4 mM	[146]
	*Bacillus subtilis*: *Escherichia coli*: *Pseudomonas fluorescens*	inhibited concentration > lmM	[147]
	*Staphylococcus aureus*	causing 100% mortality to the three tested bacteria (20 mg/L exposure 24 h).	[146]
	*Daphniamagna*; *Thamnocephalusplatyurus*; *Tetrahymena thermophila*	L (E) C_50_ values 1.1–16 gm/L	[147]
	RAW 264.7 and BEAS-2B	inhibit ROS generation, resist oxidative stress, no inflammation and cell death (25 µg/mL exposure 1–16 h)	[147]
	*Escherichia coli*	no survival above 230 mg/L; 90% survival rate (exposure 330.9 mg/L)	[148]
	human lung epithelial cells A549	40 pg/cm′ exposure 18 h inhibition 96%, after exposure 4 h DNA damaged 41%	[134]
	*Daphnia magna*; *Thamnocephalus platyurus*: *Tetrahymena thermophila*	L(E)C_50_ values 90–224 µg	[149]
	human lung epithelial cells A549	catalytic effects with ROS generating (100–200 µM exposure 60 min)	[150]
	*Caenorhabditis elegans*	LC_50_ at 82 mg/L (exposure 24 h)	[145]
	*Cupriavidus metallidurans* CH34	cell internalized and induced a drastic increase in ROS level (2 h);	[145]
	Escherichia coli MG1655	cell internalized and induced a drastic increase in ROS level (2 h);	[145]
	*Bacillus subtilis*; *Escherichia coli*; *Pseudomonas fluorescens*	inhibited 57% *B. subtilis*, 36% *E. coli*, 70% *P. fluorescens* (20 mg/L exposure 24 h)	[151]
pure metal	PC-12 cells	produce cell shrinkage, irregular membrane (5–50 g/mL exposure 24 h)	[151]
	rat liver cells BRL3A	mitochondrial perturbation (5–50 g/µL exposure 24 h)	[151]
	*Escherichia coli*	40 nm susceptibility 0.0236 mL/µg	[152]
	*Bacillus subtilis*	40 nm susceptibility 0.0622 mL/µg	[153]
	Escherichia coli ATCC 10536	shape dependent toxicity (1–100 g exposure 24 h)	[154]
	*Chlamydomonas reinhardtii*	time-dependent toxicity; NP Ag appeared to be higher than AgNO ECg3 300: 572 nM (1 h); 1 049 ± 396 nM (2 h)	[155]
	HepG2 human hepatoma cells	accelerated cell proliferation at low dose (0.5 mg/L exposure 24 h	[156]
	Zebrafish Embryos	almost 100% mortality (250 µM exposure 120 h)	[157]
	Termed HeLa: SK-Mel-28: 1929: J774A1	size-dependent toxicity (Hela cell; IC_50_ is 250 µM, 140 µM)	[158]
	Zebrafish Embryos *Escherichia coli* 33876	less than 3% mortality (250 µM exposure 120 h)	[159]
	human bronchiiall epithelial cells	oxidative stress (100–200 µM exposure 60 min)	[150]
	*Escherichia coli*	100 nm susceptibility 0.04 mL/µg	[160]
	*Bacillus subtilis*	100 nm susceptibility 0.04 mL/µg	[160]
quantum dots	WTK1 cell line	DNA damaged (2 µM exposure 2 h)	[161]
	Hela cell line, human primary, hepatocyte	cytotoxicity (0.1 mg/mL exposure 24 h)	[161]
	*Cupriavidus metallidurans* CH 34	cellular ROS level increasing and about 2.5-fold increase of 84 the cells with damaged and leaky membranes (20 nM exposure 30 min	[162]
	*Pseudomonas aeruginosa*	cell membrane damaged; intracellular ROS; a concentration threshold of 50 mg/L	[163]
	*Chlamydomonas reinhardtii*	(HS-CH-CO0-) *Sod1*, *gpx*, *cat* and *ptox2* (exposure 6 h); Transient up-regulation of genes as low as 0.1 mg/L	[162]
